# In Vitro Study of Preload Loss in Different Implant Abutment Connection Designs

**DOI:** 10.3390/ma15041392

**Published:** 2022-02-14

**Authors:** Ana Sofia Vinhas, Carlos Aroso, Filomena Salazar, Marta Relvas, Ana Cristina Braga, Blanca Ríos-Carrasco, Javier Gil, José Vicente Rios-Santos, Ana Fernández-Palacín, Mariano Herrero-Climent

**Affiliations:** 1 UNIPRO—Oral Pathology and Rehabilitation Research Unit, University Institute of Health Sciences (IUCS), CESPU, 4585-116 Gandra, Portugal; ana.vinhas@iucs.cespu.pt (A.S.V.); carlos.ribeiro@iucs.cespu.pt (C.A.); filomena.salazar@cespu.pt (F.S.); marta.relvas@iucs.cespu.pt (M.R.); 2 Department of Production and Systems Engineering, ALGORITMI Research Centre, Campus Gualtar, Minho University, 4710-057 Braga, Portugal; acb@dps.uminho.pt; 3 Department of Periodontology, School of Dentistry, Universidad de Sevilla, C/Avicena S/N, 41009 Sevilla, Spain; jvrios@us.es; 4 Technological Health Research Center, Biomaterials of the Faculties of Medicine and Dentistry, International University of Cataluña, 08034 Barcelona, Spain; xavier.gil@uic.cat; 5 Departamento de Medicina Preventiva y Salud Pública, Facultad de Medicina de Sevilla, 41009 Sevilla, Spain; afp@us.es; 6 Porto Dental Institute, 4150-518 Porto, Portugal; dr.herrero@herrerocliment.com

**Keywords:** implant abutment connections, preload loss, tightening torque, retightening, cyclic loading

## Abstract

The stability and integrity of the abutment-implant connection, by means of a screw, is fallible from the moment the prosthetic elements are joined and is dependent on the applied preload, wear of the components and function. One of the main causes of screw loosening is the loss of preload. The loosening of the screw-abutment can cause complications such as screw fracture, marginal gap, peri-implantitis, bacterial microleakage, loosening of the crown and discomfort of the patient. It is also reported that loosening of the screw/abutment may lead to a failure of osseointegration. It is necessary to evaluate and quantify, with in vitro studies, the torque loss before and after loading in the different connections. **Aim:** evaluate the influence of implant- abutment connection design in torque maintenance after single tightening, multiple tightening and multiple tightening followed by mechanical cycling. **Materials and Methods**: 180 Klockner implants divided in 4 groups: 15 SK2 external connection, 25 Ncm tightening torque; 15 KL external connection, 30 Ncm tightening torque; 15 Vega internal connection, 25 Ncm tightening torque; 15 Essential internal connection, 30 Ncm tightening torque. In each group removal torque values (RTV) were evaluated with a digital torque meter, in 3 distinct phases: after one single tightening, 10 multiple tightenings and 10 multiple tightenings and cyclic loading (500 N × 1000 cycles). **Results:** After one single tightening, and for all connections, RTV were lower than those of insertion, but only for Essential and Vega internal connections this result was statistically significant. After multiple tightening, RTV were significantly lower in all connections. After repeated tightening followed by cyclic loading, mean RTV were significantly lower, when compared to insertion torque. The multiple tightening technique resulted in higher RTV than the single tightening technique, except for Vega implant. The multiple tightening followed by cyclic load, compared to the other phases, was the one that generated the lowest RTV, for all connections. **Conclusions:** The connection design, in our study, did not seem to influence the maintenance of preload. Loading influenced the loss of preload, in the sense that significantly decreased the removal torque values. The multiple re-tightening technique resulted in higher removal torque values than the single tightening technique. Clinically, our results recommend to retighten retaining screws, a few minutes after insertion.

## 1. Introduction

Over the past 30 years, clinical evidence has shown excellent long-term results in implant osseointegration, with high success rates in the order of 90% [1,2]. However, this predictable treatment requires a dynamic balance between mechanical and biological factors. One of the disadvantages of implant rehabilitation is mechanical complications, which can occur during the clinical life of the implant. In the tightening of the components of the connection, a tension occurs with a consequent compression between the structures of the union. The most frequent complication of implant-supported restorations is the loosening of the fixing screw, with an estimated annual rate of 2.1% [3]. The estimated rates are 10.4% and 20.8% over 5 and 10 years, respectively [4]. Different manufacturers recommend a preload torque between 10 and 35 Ncm, depending on the manufacturing material of the screw and the design of abutment-implant connection [5].

Preload is the force that is generated when the screw is tightened using a given torque [5,6,7]. Torque is defined as the movement produced by applying tangential force to the screw, usually inserted using a torque wrench, and expressed in newton centimeters (Ncm). When applying the preload to a screw, the connected elements are kept in compression, and the screw receives small impacts because most of the load is absorbed by the components of the implant–abutment joint. [8] One of the most common reason of screw loosening is the “loss of preload”. Only 10% of the initial torque is transformed into preload, whereas the remaining 90% is used to overcome the friction between the surface irregularities [5,6,7]. In the tightening of the components of the connection, tension occurs with a consequent compression between the structures of the joint. From a clinical perspective, the loosening of the screw is greater in external connections than in internal connections, with an incidence of loose screws of 38% in external hexagon systems [9,10]. Mechanical complications, such as loosening and fracture of the prosthetic abutment fixing screw, have been associated with the implant-abutment connection type [11]. This connection is the weakest point of the complex, since it must be resistant to occlusal forces, prevent micro-movement and minimize bacterial leakage [12]. The microgap seems to play a key role in the process of bacterial colonization of the fixture-abutment interface. This area is widely studied in literature in terms of microgap reduction and improvement of the implant-abutment connection [13]. Several authors analyzed the existing microgap describing values between 10 and 135 µm [14,15]. Preload loss can favor the occurrence of implant-abutment interface misfit, there is no implant connection system that provides an implant-abutment interface seal against bacterial penetration [16]. The existing space between fixture and abutments remains a crucial area in bacterial colonization as a starting point for the peri-implant marginal bone loss around the fixture [13].

In recent years, the geometries of implant connections have been developed with different biological and aesthetic characteristics. The first dental implants were comprised of an external connection system with internal connection implants appearing later. External connections usually have an external hexagon on the implant platform (0.7 mm-high hexagon), while internal connections can be divided into internal hexagons, internal octagons, and Cone Morse connections [17].

It is necessary to evaluate and quantify, with in vitro studies, the screw joint stability and the integrity in the structure of the components, before and after loading, in the different connections.

The aim of this in vitro study was to evaluate the influence of implant- abutment connection design, in preload loss, after single tightening, multiple tightening and multiple tightening followed by mechanical cycling.

## 2. Materials and Methods

**Figure 1:**
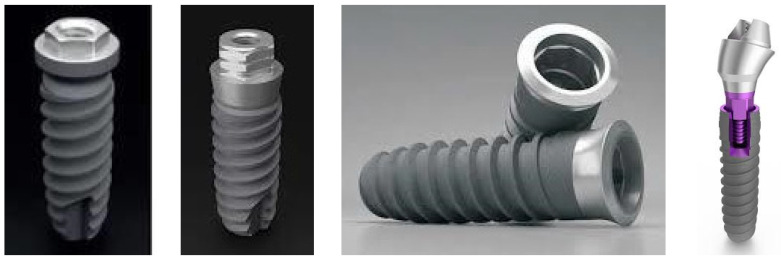
Klockner Implants included KL^®^, SK2^®^, Essential^®^ and Vega^®^.

**Figure 2:**
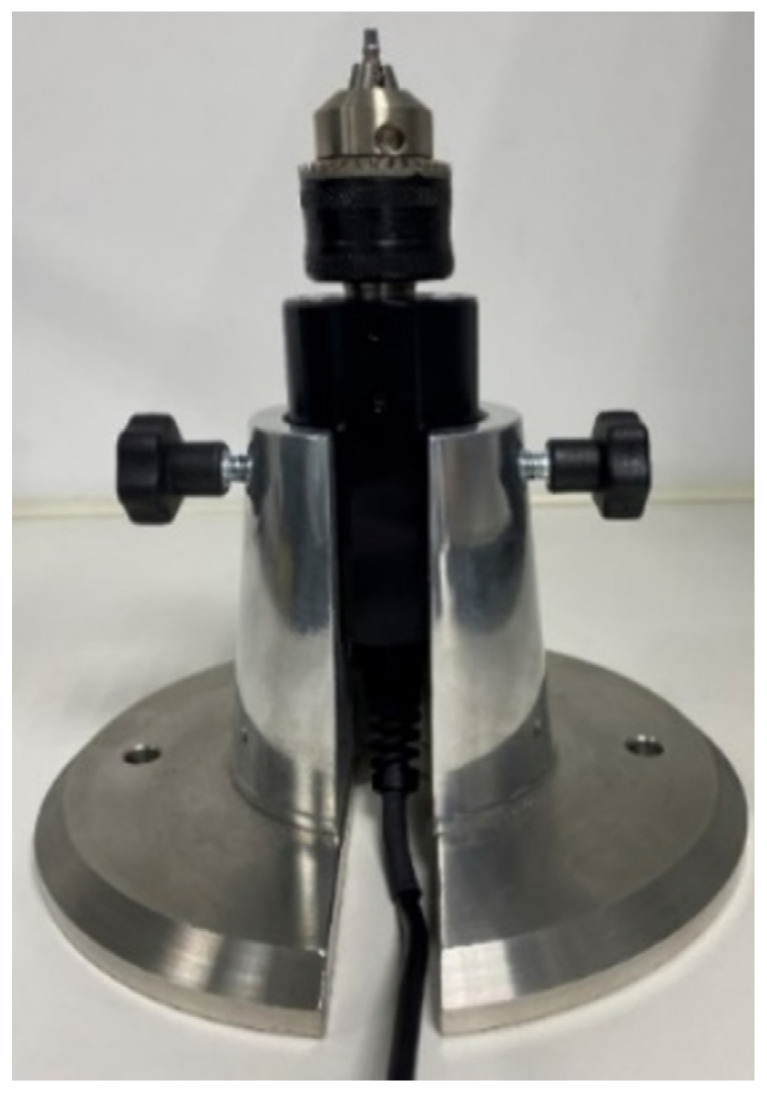
Metallic Suport holding the chuck.

This blinded design in vitro study, evaluated 4 systems of Klockner implants (SOADCO-Andorra) 2 internal connection systems: VEGA^®^ and ESSENTIAL^®^ and 2 external connection systems: SK2^®^ and KL^®^ (Figure 1). The implants were 4mm diameter and 12 mm length, and the insertion torque of 30 Ncm (Essential and KL) and 25 Ncm (SK2 and Vega) was the recommended by the manufacturer. Fifteen implants of each type were placed on a metallic support (Figure 2) leaving the most coronal portion 2 mm outside the device, which allowed the installation of the implants in the machine responsible for the application of the cyclic load, as well as the access for the digital torque meter.

### 2.1. KL 0.7^®^ Implant

External connection implant made of commercially pure titanium grade III and IV. KL^®^ is an implant with a slightly ogival thread with two sections at the tip to facilitate surgical insertion, presents a double spire, with an advance step of 2.2 mm which allows to reduce surgical times. The KL implants^®^ present as connection a hexagon of 0.7 mm height that allows to raise the gap of connection with respect to the bone crest.

### 2.2. SK2^®^ Implant

External connection implant, with a hexagon 1.8 mm high, made of commercially pure grade III titanium. They have a machined neck that allows to raise the connection gap with respect to the bone crest, the surface roughness favors the sealing of the soft tissues and the surface treatment allows greater contact surface. The apical zone emerges with a programmed conicity that facilitates the insertion of the implant, the central area of parallel walls offers a great primary stability, the conical cervical area ends in the maximum diameter of 4.2 mm of the shoulder of the implant platform.

### 2.3. Essential^®^ Implant Cone 0.7 and 1.5

System of internal connection implant made of commercially pure grade III titanium. The Essential^®^ implant is a double-spire implant and easy insertion that makes it possible to address compromised anatomical areas thanks to the atraumatic design of the apical area and the different lengths available. Its design at the cervical level is planned for placement following the Semi Submerged Technique generating an optimal biological seal that prevents bone resorption caused by bacterial infiltration through the connection gap.

### 2.4. VEGA^®^ Implant

VEGA^®^ is the system of implants of internal connection specially thought and designed for the treatment of all surgical and prosthetic solutions that require working with implants at the bone level. Its main indication is rehabilitation in aesthetic areas, thanks to its design that allows the maintenance of the crestal bone and guarantees the correct sealing of the peri-implant soft tissues. The VEGA^®^ implant is made of the new OPTIMUM^®^ titanium. The development and application of the new titanium has made it possible to increase the yield strength and improve mechanical properties in 64% of the entire range of VEGA implants^®^. The conical design of the implant in its most coronal portion allows a better distribution of loads to the adjacent bone tissue. The micro grooves dissipate stress on the crestal portion, preventing bone loss when the implants are loaded, helping to maintain the bone level.

45 Implants of each platform/45 straight titanium abutments/45 titanium screws were distributed in 3 groups of 15 each to use in the 3 different phases of the study: (Table 1).

Phase I: assessment of preload after single tightening: The abutment was connected to the implant; the screw was fixed with the torque recommended by the manufacturer. This operation was performed only once. Removal torque value was evaluated after 1 min with a digital torque meter.

Phase II: assessment of preload after multiple tightening: The abutment was connected to the implant; the screw was fixed with the torque recommended by the manufacturer. This operation was performed 10 times with a time interval of 15 s between each tightening. Removal torque value was evaluated after 1 min with a digital torque meter.

Phase III: assessment of preload after multiple tightening and loading. The abutment was connected to the implant, the screw was fixed with the torque recommended by the manufacturer. This operation was performed 10 times with a time interval of 15 s between each tightening. Removal torque value was evaluated, with a digital torque meter, after applying a load of 500 N for 1000 cycles.

### 2.5. Equipment Used

Metallic support: to achieve chuck stability by tightening and retightening the screw that attaches the abutment to the implant, we designed and manufactured a metallic support, in stainless steel, to hold the chuck and the samples to allow torque measurement. (Company Jovicar, Braga, Portugal) (Figure 2).

**Figure 3:**
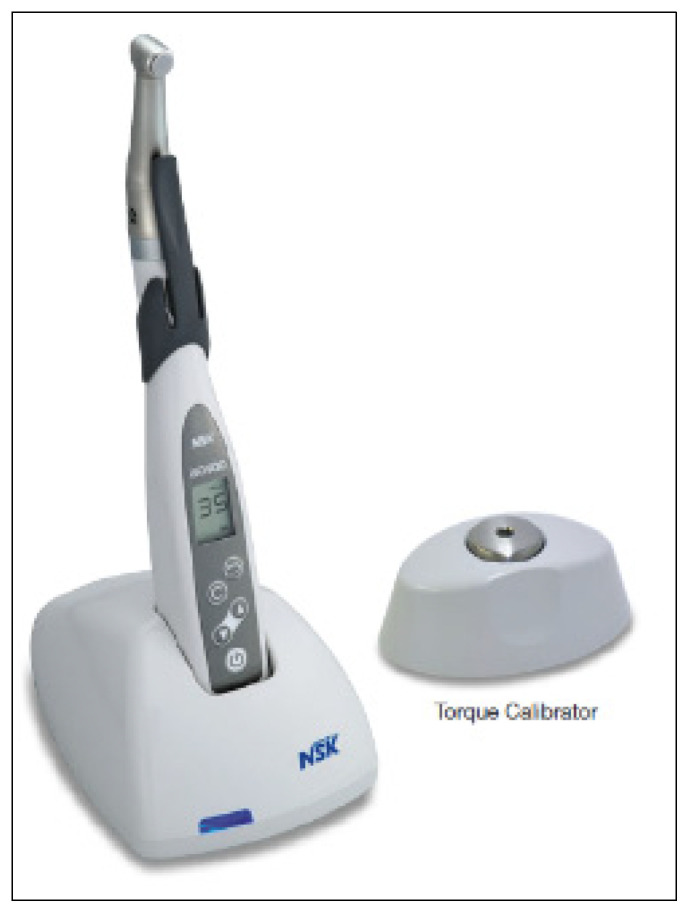
Cordless Prosthodontic Screwdriver.

Cordless Prosthodontic Screwdriver with Torque Calibration System (TSC): to connect the abutments to the implants was used a cordless screwdriver, with torque calibration system for prosthodontic screws fixing procedures (Company NSK^®^ model iSD900^®^, Tokyo, Japan) (Figure 3).

**Figure 4:**
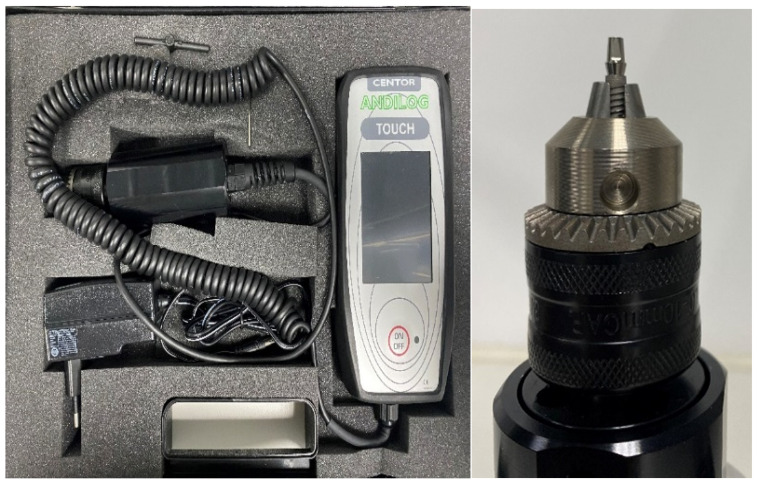
Digital Torque Gauge with the chuck to hold the samples.

Manual Torque Gauge: a digital torque meter was used to evaluate Removal Torque values in the 3 phases, with its handle screwdriver sensor that allows an efficient and easy variety torque measurements. (Company Andilog Technologies, Centor Touch Star model TH^®^, Vitrolles, France) (Figure 4).

**Figure 5:**
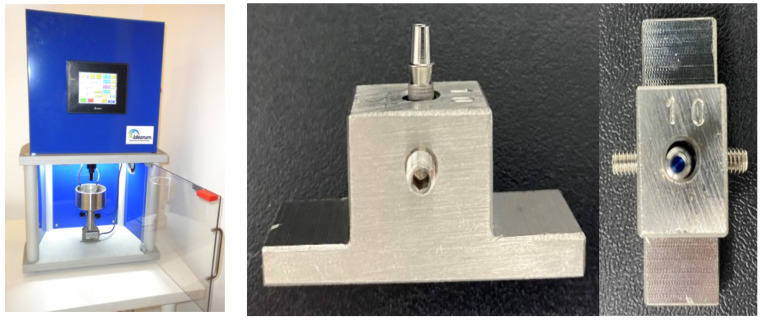
Fatigue testing machine and metallic supports holding a sample to be tested.

Fatigue Testing Machine: the loading cycles were carried out by a fatigue machine, CS Dental Testing Machine [18,19,20] with an axial load of 500 N for 1000 cycles. (Company Idearum, Model ID1-BAD, Barcelona, Spain) The samples were placed in metallic supports, designed by us, in a hole 10 mm deep, which allow the insertion of the implants to be tested, with exposition of 2 mm of the most coronal portion. (Company Jovicar, Braga, Portugal) (Figure 5).

### 2.6. Statistical Analysis

Sample size: using the data obtained by Al-Otaibi [21], the N Query advisor program was used, obtaining a minimum size of 33 per group for a significance level of *p* < 0.05, 3 groups (unitary, multiple and cyclical) a variance of the means calculated from the ‘detorque’, with a power of 80%.

The Statistical analysis was done using the IBM^®^ SPSS^®^ (Statistical Package for Social Sciences) Statistics version 27.0, given the nature of the variables involved the analysis consisted of:

- in the descriptive study of the data—qualitative and quantitative variables (bar charts, circulars, frequency tables, wire box charts).
- evaluation of data distribution—Shapiro-Wilks’s test (SW) to test the normality adjustment of the data (for continuous variables) and/or graphical methods (P-P plot).
- in the comparative study—t-student comparison tests (for two independent samples) or if the normality assumption is not met by its non-parametric equivalent, if the conditions of the Central Limit Theorem (large samples) are not applicable. For more than two normal independent samples, the ANOVA methodology or its non-parametric equivalent, Kruskal-Walli’s test, was used.
- in the comparative study of pairs—t-student test for 2 paired samples (when comparing two torque measurements) for the pairs of measurements evaluated. When the normal conditions were not met, the Wilcoxon test (W) was used.
- in the comparative study over time—ANOVA tests with repeated measurements with k evaluation levels (k = 10 different times).
- In the comparative study of two factors (binding and phase)—ANOVA two way to evaluate the differences in tightening and untightening torque.

The decision rule used is to detect statistically significant evidence for probability values below 0.05.

## 3. Results

Phase 1—intended to evaluate the loss of preload after one single tightening, and it has been verified that, for all samples, removal torque values (MU) were lower than those of insertion (MT). It has also been found that only for Essential and Vega connections, at maximum tightening, are not guaranteed the normality conditions (*p* < 0.05). This result revealed that removal torque value was significantly lower than the tightening torque for Essential and Vega, the internal connections.

Phase 2—pretended to access the loss of preload after multiple tightening, and the main results are summarized in Table 2. Removal torque values (UM) were significantly lower in all connections. Shapiro-Wilks’s test was performed to assess normality for the variables maximum tightening and untightening, having verified that only for Vega connection, in untightening, is not guaranteed the normality condition (*p* < 0.05) (Table 3).

Phase 3—assessed loss of preload after multiple tightenings and cyclic loading. These results revealed that removal torque values (MU) are significantly lower than the tightening torque for all connections (Table 2). The variation in the average load was also verified in each of the connection groups. For this, the ANOVA procedure was performed, obtaining F (3;56) = 20.122 and *p*-value < 0.05, which allowed us to conclude that there were significant differences in the mean value of removal torque, after cycling load, in each connection.

**Figure 6:**
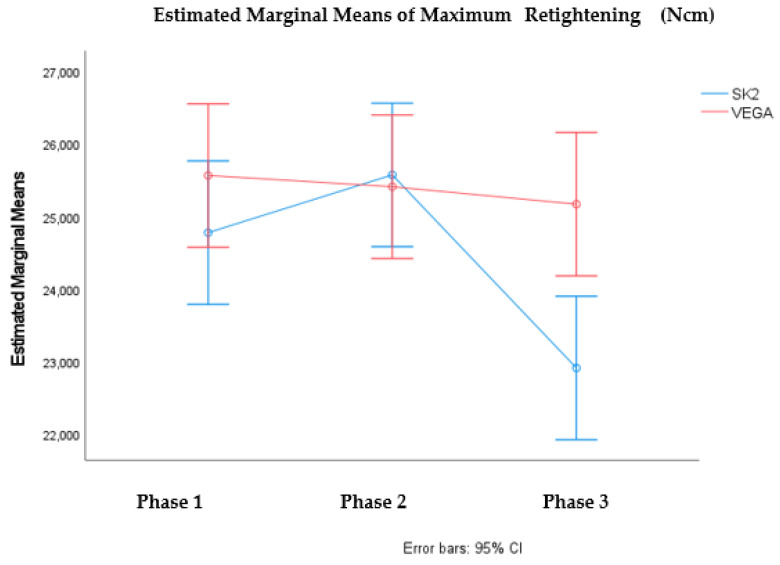
Profile graph for the average value of Maximum Untightening according to phases and connection (SK2 vs. VEGA).

**Figure 7:**
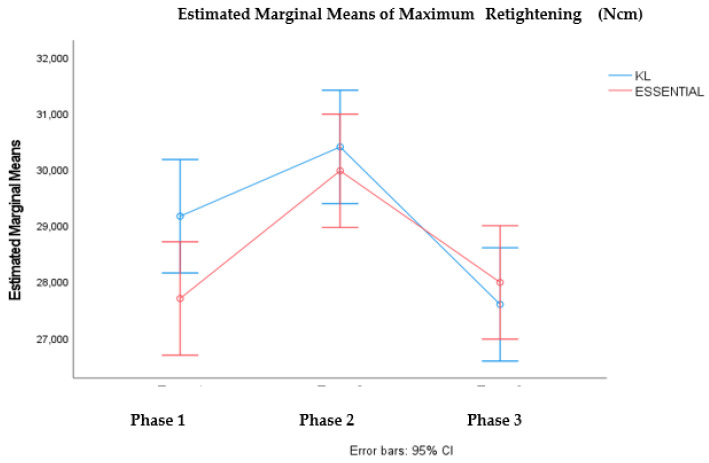
Profile graph for the average value of Maximum Untightening according to phases and connection (KL vs. ESSENTIAL).

It was verified statistically significant differences in the mean value of maximum untightening, in the different phases considered F (2; 84) = 4.697, *p* < value 0.05, and also between the mean values of the maximum untightening of the SK2 and VEGA connections F (1;84) = 5.598, *p*-value < 0.05). Multiple comparation tests for the phases showed significant differences between phase 2 and 3. These results are illustrated in the graph in Figure 6. It was also verified that there are statistically significant differences in the mean value of the maximum untightening in KL and Essential, in the different phases considered F (2; 84) = 11.919, *p* < 0.05. Multiple comparation tests showed significant differences between phase 2 and phase 1 and phase 2 and phase 3. These results are illustrated in the graph in Figure 7.

Comparing the 3 phases with the 2 types of connection and the theoretical insertion torque: Sk2 external and Vega internal (25 Ncm); KL external and Essential internal (30 Ncm), statistically SK2 and Vega differ from each other in the 3 phases, and Essential and KL have similar behavior.

## 4. Discussion

Phase I: assessment of preload after single tightening:

In our study, the results of preload maintenance, after single tightening, in the 4 evaluated implants (2 external connection-KL and SK2; 2 internal connection- Essential and Vega), are similar to numerous studies previously conducted. Regardless of the type of connection, the average value of the removal torque is less than the initial tightening value. The results of Al-Otaibi et al. and Jorge et al. and [21,22] corroborate ours, they have found that all removal torque values were lower than the initial insertion torque in external hexagonal and in internal connection The loss of torque, a few minutes after the application of the preload is expected and can be explained by a phenomenon known as sedimentation effect [9,23]. When torque is applied energy is dissipated in smoothing mating surfaces, reducing elongation of the screw. Embedment relaxation of mating surfaces results from the fact that, because of machining microroughness, no 2 surfaces are in complete contact with one another. According to Breeding et al. [24], deformation and flow of components can reduce torque by 2% to 10% in the first moments after tightening. This explains why, clinically, it is recommended to tighten the retention screw again 10 min after applying the initial torque. Our study has shown that, for internal connection implants, the removal torque is significantly lower than the insertion torque (Essential-30,497/27,692 *p* < 0.001; Vega- 26,496/25,562 *p* < 0.016). Comparing implants with the same theoretical torque of single tightening (25 Ncmm-Vega and SK2; 30 Ncm-Essential and KL) and respective untightening there were no significant differences between internal and external connections.

Phase II: assessment of preload after multiple tightening:

The torque value evaluated, after screw loosening, is an indirect measurement of the remaining preload. The objective of this phase was to evaluate the torque maintenance of the retention screws, after repeated cycles of tightening/untightening of the screws. In the literature, the mean values of the removal torque were found to be lower than the insertion torque values. This fact can be explained due to the phenomenon previously described as sedimentation effect [25,26]. The loss of torque, after multiple tightening, demonstrates that some of the insertion torque, used to generate the preload, is lost, even before function. Clinically, current results indicate that retaining screws should be readjusted after 3 min of insertion, before masticatory loading occurs. When torque is first applied, some of the torque is used to flatten the surface roughness in the internal threads of the implant and the surface of the screw. The second application of torque generates the desired preload, and this may explain why the multiple re-tightening technique resulted in higher removal torque values than the single tightening technique [27]. In effect our results confirm these findings, except for the Vega implant that obtained a lower average value of removal torque with multiple tightenings (25.40 Ncm), compared to single tightening technique (25.56 Ncm). The study by Kim et al. [28] supports these findings and confirms that it should be taken into account that the loss of preload, due to the sedimentation effect, can lead to loosening of the screws. Although several authors have recommended re-tightening the screws after a predetermined interval, to overcome the problem of preload loss [5,26,29,30,31,32,33], others suggest that repeated tightening has little or no effect, and it can even flatten more the contact surfaces, and cause a significant loss of preload [27,34,35,36,37,38]. In our study, for the 4 implants included with insertion torque of 30 Ncm (Essential and KL) and 25 Ncm (SK2 and Vega) there is significant variation in the mean torque values after 10 tightenings, in the sense that it increases significantly over time.

Phase III: assessment of preload after multiple tightening and loading.

Cyclic load forces, during physiological function, that do not exceed the maximum force of the implant-abutment connection can gradually loosen the implant-abutment connection or cause it to fail, due to fatigue. The critical reason for the loosening of the abutment-implant connection is the loss of preload on the screw, and the consequent tightening or fatigue failure of the screw material. Removal torque values have been used as a preload measurement in numerous studies to assess interface stability after fatigue testing [39]. The objective of this phase was to investigate whether repeated tightening and untightening of the screw, and the application of cyclic loading, would affect the removal torque value of the screw. In our study, the multiple tightening followed by cyclic loading, compared to other phases, was the moment that generated lower removal torque means.

We identified 2 studies (Cashman et al. and Arshad et al.) [37,40] that investigated if repeated screw joint tightening would affect the post-fatigue abutment screw removal torque. Arshad et al. results indicated that removal torque values, after loading, were considerably lower, than the insertion torque, in the conical hexagon connection. These results corroborate previous studies, which reported that all screw types showed decreased preload values with repeated tightening. However, Cashman et al., did not reveal a significant loss of removal torque values post fatigue loading. The differences in chemical composition, manufacturing, surface implant treatment, number of loading cycles, direction and loading values may explain this result.

Our study also reported that all connections show some decrease in preload with repeated tightening, followed by cyclic loading. In this phase, for KL, SK2, Essential and Vega implants, the mean removal torque values were significantly lower when compared to insertion torques. When comparing the means of tightening and untightening torque in implants with the same theoretical insertion torque, the Sk2 and Vega implants (25 Ncm) have shown significant differences (28.27 Ncm/22.90 Ncm; 27.21 Ncm/25.16 Ncm respectively). In contrast KL and Essential, implants, with theoretical insertion torque of 30 Ncm, presented similar means of removal torque. Many authors indicate that external connection systems exhibit better fatigue behavior due to the connection design [41,42]. Based on these conclusions, we identified the studies of Shin et al. and Gil et al. [43,44]. In our phase 3 study, in relation to implant design, no difference was found between the behavior of the internal connection and the external hexagonal implant systems. The studies of Piermatti et al., Tsuruta et al., and Tsuge et al. [7,34,45], as in our study, did not verified superiority fatigue behavior in any of the evaluated connection designs (internal, external and conical).

There were some limitations to this study inherent to the in vitro condition. The oral environment and conditions could not be exactly simulated, thus the results should be interpreted with caution and validated in a clinical condition.

## 5. Conclusions

The results of the available studies concerning the maintenance of preload have presented diversity, which can be explained by the variety of methodologies, the different values and directions of the applied load, the number of load cycles, the different fatigue machines and the number of samples evaluated. Some studies compared the different implant designs available, and others included only one type of connection system. The connection design, in our study, did not seem to influence the maintenance of preload. Loading influenced the loss of preload, in the sense that significantly decreases the removal torque values. The multiple tightening technique resulted in higher removal torque values than the single tightening technique. When torque is first applied, some of the torque is used to flatten the surface roughness of the components. Clinically, our results recommend to retighten retaining screws, after insertion (3 to 10 min), before masticatory loading occurs, and this second application of torque will generate the desired preload.

## Figures and Tables

**Table 1:** Total Samples used (N = 180).

Connection System	Single Tightening	Multiple Tightening	Cyclic Loading
Vega (45)	15	15	15
Essential (45)	15	15	15
SK2 (45)	15	15	15
KL (45)	15	15	15

**Table 2:** Comparison of the Means of Maximum Tightening (MT) and Maximum Untightening (MU) of the 4 implants in the 3 phases of the study. * Significant result (*p* < 0.05).

PHASE		KL	SK2	ESSENTIAL	VEGA
1. Single tightening	MT	29.74 Ncm	26.18 Ncm	30.49 Ncm *	26.49 Ncm *
	MU	29.16 Ncm	24.77 Ncm	27.69 Ncm *	25.56 Ncm *
2. Multiple tightening	MT	32.46 Ncm *	27.68 Ncm *	32.69 Ncm *	27.68 Ncm *
	MU	30.39 Ncm *	25.57 Ncm *	29.97 Ncm *	25.40 Ncm *
3. Multiple tightening + loading	MT	32.45 Ncm *	28.27 Ncm *	32.24 Ncm *	27.21 Ncm *
	MU	27.58 Ncm *	22.90 Ncm *	27.98 Ncm *	25.16 Ncm *

**Table 3:** Normality Test Shapiro-Wilks—Phase 2.

PHASE	Group	W ^†^	Df ^†^	*p* ^†^
Maximum tightening (Ncm)	KL	0.922	15	0.408
	SK2	0.923	15	0.216
	ESSENTIAL	0.924	15	0.223
	VEGA	0.946	15	0.469
Untightening (Ncm)	KL	0.987	15	0.997
	SK2	0.930	15	0.277
	ESSENTIAL	0.916	15	0.168
	VEGA	0.850	15	0.018

**^†^** W—Shapiro-Wilks Statistic, df—degrees of freedom, *p*—probability value.

## Data Availability

The data presented in this study are available on request from the corresponding author.
